# Lest We Forget

**Published:** 1916-02

**Authors:** J. Clarence Johnson

**Affiliations:** Atlanta, Ga.


### Journal-Record of Medicine

Successor to Atlanta Medical and Surgical Journal, Established 1855
and Southern Medical Record, Established 1870
**OWNED BY THE ATLANTA MEDICAL JOURNAL COMPANY**
PUBLISHED MONTHLY

*Official Organ Fulton County Medical Society, State Examing Board, Atlanta, Birmingham and Atlantic Railroad Surgeon's Association, Chattahoochee Valley Medical and Surgical Association, Etc.*

RICHARD R. DALY, M. D., Editor
BERNARD WOLFF, M. D., Supervising Editor
A. W. STIRLING, M. D., C. M., D. P. H.; J. S. HURT, B. Ph., M.D.
GEO. M. NILES, M. D., W. J. LOVE, M. D., (Ala.); Associate Editors
E. W. ALLEN, Business Manager

**COLLABORATORS**

W. F. WESTMORELAND, M. D., General Surgery
F. W. McRAE, M. D., Abdominal Surgery
ALLEN H. BUNCE, Medical Societies
E. B. BLOCK, M. D., Diseases of the Nervous System
MICHAEL HOKE, M. D., Orthopedic Surgery
CYRUS W. STRICKLER, M. D., Legal Medicine and Medical Legislation
E. C. DAVIS, A. B., M. D., Obstetrics
E. G. JONES, A. B., M. D., Gynecology
ALLEN H. BUNCE, M. D., Pathology and Bacteriology
R. T. DORSEY, Jr., B. S., M. D., Medicine
L. M. GAINES, A. B., M. D., Internal Medicine
GEO. C. MIZELL, M. D., Diseases of the Stomach and Intestines
L. B. CLARKE, M. D., Pediatrics
EDGAR PAULLIN, M. D., Opsonic Medicine
THEODORE TOEPEL, M. D., Mechano Therapy
R. R. DALY, M. D., Diseases of the Eye, Ear, Nose and Throat
BERNARD WOLFF, M. D., Diseases of the Skin
E. G. BALLENGER, M. D., Diseases of the Genito-Urinary Organs

VOL. LXII. ATLANTA, GA., FEBRUARY, 1916. NO. 11

### "LEST WE FORGET."

By J. Clarence Johnson, M. D., Atlanta, Ga.

In this day of Militarism any one who dissents from the existing order of things is apt to be counted a disturber of the peace. I hope to avoid this distinction while I try to say a few words about Medical Preparedness,—not for war alone, but for all times, and for all circumstances. I would not speak carelessly of this, however unimportant and ineffective my opinions may be, because I think that it is a subject upon which venture should be made with soberness and all good reason. I ask you, therefore, to bear with me in my efforts to first place the matter in its proper light.

Hardly any one of us has failed to notice the recent changes in the manner of teaching and in the practice of medicine and surgery. We have felt a difference in the attitude of the laity towards the profession at large, and in some particulars individually,—we are conscious of a newness in the active relations of the members of the profession. We have witnessed the growing paternalism of the government towards medical work and the establishment of foundatinos for research· We have daily evidences by word of mouth and by published report of the greater freedom exercised in professional affairs by medical men and the laity alike and of better opportunities for enlarging this freedom. In all this rapid advance something of necessity has been left behind and something lost.

While the practice and the art of Medicine have moved away from their long established bases, the science of medicine has remained the same. We know more of the science, and know it better. Everything which has increased our knowledge of the science and has at the same time simplified the art and practice of medicine has been for betterment. Everything which has intensified our knowledge of the science and has at the same time complicated the art and practice has been a hindrance,—for the art and practice of medicine are but the application of the science. In other words, to that extent to which all the changes wrought have multiplied the burdens of the masses of the profession without supplying them with ready means to bear them, has general effectiveness been diminished. These facts become more pertinent when we consider that there are now less than half the matriculates in Medical Colleges than were enrolled 15 years ago. With superior advantages it is to be supposed that the reduction in number has been somewhat counterbalanced by increase in individual fitness. Nevertheless there is a limit to what any man can learn, and to what any man can do, and general results cannot be rightly measured by what is done by individual or a class, but by what is done by all. By these results also is the average efficiency determined. There will always be some men in medicine and in surgery who know more than others and who do more than others. And such men will be regarded as leaders of thought and action. The world will always and should respect the opinion of those who having given proof of their wisdom and of their sincerity, speak of universal interests and of universal good. But no one in infallible and to save us from the evils of mistake in the Book of all Knowledge we are advised that in the mouths of many witnesses shall the truth be established. Regardless of its character or of its source a fact should be self evident or capable of proof before it is accepted as a fact. No man can be the author of a truth or authority in law. One may discover the truth,—may be authority on the law, or have authority by the law,—that is all. Law is authority within itself and unto itself. Law has its own standards, measured but not made by men. These standards are fixed by the principles out of which the law is evolved. There is no revision of law which sacrifices these principles. An act involving the law is adjudged by these principles. An opinion handed down is the interpretation of these principles.

There is no essential difference between the principles of one law and another. The principles of civic law and scientific law are alike founded upon elementary truth. The principles in medicine are as fixed and unchangeable as are the forces of physics, chemistry, physiology, which are represented in all action and in all forms of organic life. There is no difference between these forces and principles in health and in disease and there is no appeal from the laws which govern them. Yet in the practice and in the teaching of Medicine opinions are being handed down from some high places which do not take these facts into full account. These opinions are accepted by some and are applied to action. It matters not whether the error is due to ignorance or to overzeal, the result is the same. The verdict in the case is not based upon scientific facts, but chiefly upon physical evidence alone. The evidence bears the same relation to the disease that sound does to an explosion. It does not show what has caused the explosion or what the consequences are,—You cannot make a complete and seldom a correct diagnosis on a cadaver, any more than you can tell what is the trouble with an engine while it is standing still. You can detect and describe morbid anatomy, but morbid anatomy is the result of disease itself. Disease means pathological action and action means function and function means life.

Failure to admit and keep in mind these facts may sometimes prove costly to the profession and the public. Let us apply them.

In a recent issue of a northern paper, one of the most impartial and widely read, there was an editorial comment upon the reports of two of the leading hospitals of the country. In this editorial attention was called to statements in these reports showing that according to post-morten examinations made in these hospitals diagnosis had been wrong in 50 and 60 per cent. of the cases respectively. When we consider the source of these reports and their significanc is not surprising that a newspaper should find in them matter for discussion. If the reports are true the facts as stated are startling. There is no doubt of their being accepted as true by the public and by some of the profession, and there is no doubt of the effect of these reports on public confidence in professional skill and upon the confidence of the profession in its own ability.

It will not perhaps be remembered that only a small percentage of patients admitted to a hospital dies. It might be inferred that some of those who die, do so because of mistaken diagnosis. This may be true. It may also be inferred that an equal per cent. of those who get well do so because of correct diagnosis. But this is not always the belief. There is a popular fallacy that the majority of sick people would get well without medical treatment. There are some of the profession who encourage this sense of security. Apparently it is well founded, but there is a vast different between a house catching fire and burning down. There is a vast difference between recovering from rheumatism and recovering from the effects of rheumatism. There is a vast difference between suspension of alcoholics and suspending the effects of alcoholics. Nevertheless the impression is easily left that while 50 per cent. of autopsies show error in diagnosis, a greater per cent. of recoveries have not had correct diagnosis. In the light of modern means of investigation, laboratory, X-ray, etc., this does not reflect great credit upon the medical profession. I do not believe that in Atlanta 50 per cent. of diagnosis of those who die or of those who live are mistaken and I suspect that these reports were based upon technical and not upon practical findings. The possibility of the confusion and the effect of all this has its foundation in one fundamental error which we can easily make. That of emphasizing the importance of position to the neglect of conditions,—considering size of such account that we overlook function,—seeking to name the disease instead of studying its features, endeavoring to prove a fact by the evolution of a theory instead of proving a theory by the application of a fact—withal, forgetting the alphabet of diagnosis found in the principles of Anatomy, Physiology, Chemistry, and Physics. Diseases in their character and course have much in common with human life which we have been informed, is just one thing after another. The sooner the public realizes the causative relation which diseases bear to each other the sooner will we reach the goal of preventive medicine, and some of the profession can afford to have part in this realization.

A people or a nation do what they are taught. Education is the source of all power, whether that education be right or wrong, whether that power be for good or evil.

The business of medical education is not confined to colleges, and the students of medicine are not always graduates or matriculates. There is no adventure more fascinating to the lay mind than that which carries them into the mysteries of the human body, about which under our present system they can know but little and of which they can understand less. It matters not the quality of this education its scope is being widened and its influence is growing stronger. People who are not trained to think for themselves are willing to have opinions made for them, just as they are willing to be led without knowing where they are going. For this reason the most blatent quack or patent medicine vender has right of way into public confidence. Besides some of the leading journals sensing the curious appetites of the masses are beginning to feed them with quasi medical articles from the pens of professional men supposed to be in good standing. In point of fact, there is no more moral reason why a doctor should not use the press to make his opinion known than a lawyer, a preacher, or a poet. But there is a diet unfit for babes,—and a Biblical injunction about what not to do with pearls. The time is not yet when the profession can wisely or safely disseminate certain kinds of knowledge for general use. It is not the part of a physician to pervert the moral forces of mankind. And the old saying that a little learning is a dangerous thing is as true as it ever was. Conservative advance along public lines can be made by the profession only when we have fixed a standard for ourselves and when this standard represents what is true in science and right in practice. This standard should also indicate the educational quality and power of the profession itself.

Doctors are expected to disagree and it is not phenomenal that the laity are often disconcerted and in doubt about those they know, and have superstitious faith in those whom they do not know.

In a court of law a doctor as an expert witness often stands convicted of ignorance before he has said a word, for the simple reason that the profession has encouraged the presumption that nothing is positive and nothing sure. Even if he be suspected of a willingness to tell the truth, he is not credited with the means of doing so. By common consent and public appraisal he is judged not by what he really knows and says, but by comparison of what he says with the opinion of some self-constituted and remote authority who is just as much doubted at his end of the line. Therefore, while he is sworn to 'tell the truth, the whole truth and nothing but the truth, so help him—, usually he is not allowed to tell half of it. Notwithstanding the law not only entitles him to tell it, but demands of him under penalty that he does tell it all. This imposition and reflection upon the profession has its source in the conditions which I am trying to make clear tonight. In a word, to a lack of standardization—a law which all are supposed to know and of which and by which we only as physicians, have the right to speak with absolute authority.

There is no rightful place for much that militates against the professional and personal welfare of medical men and all the fads and fancies which have taken hold upon the laity are but the weeds which have grown upon a soil neglected, or left to care for itself, and from which, perhaps, some of the stronger elements may have been removed.

Nothing is made stronger by stretching. As a rule resistance increases with density. So the ranks of our profession do not grow stronger by standing apart, or by dividing into camps in our works, or in our ideas. I would not indulge conceit, and I do not claim prophetic vision, but times have changed wonderfully during the past few months, and the great revolution which is sweeping other institutions from their bases will not leave medicine untouched, either as a science, a practice, or a means of livelihood. New adjustments must be made in every phase of life, and we will have to fit into the plan of reorganization and repair. The forces which have so far hindered our progress may have new direction. The forces which have favored it may have new impetus. To overcome the evils that exist, and to accomplish the good which is possible we must look to education and unity,—the only dependable power that we have.

Doctors educate each other. There are men who by virtue of untiring energy and great skill attain to an eminence which first attracts admiration and then commands discipleship. They are the ones who speak the last word in that department of science which they represent. Therefore, it is of immeasurable importance that this word be according to the law. If it is not the error of its adoption cannot be amended. You can restore personal liberty, but you cannot restore life or a part which has been removed. You cannot gather again the seeds which have been scattered to the four corners of the earth,—which have taken root and grown to maturity, and have reproduced their kind. The channels of our education are not too well guarded against the evil of overzeal on the one hand and of bias and indifference on the other. Witness the magnification of intestinal stasis and the quick resort to radical measures for its relief. Witness the absurd doctrine promulgated by some of the leading surgeons of the country and endorsed by others, that more than 90 per cent. of Gastro-intestinal diseases are purely surgical.

Now, Sam Jones used to say that a hit dog yelps. This is so if he is a sure enough dog. It has also been said that its a poor dog that won't wag his own tail. I have never been informed whether this means that, the dog is too poor to wag his tail, or that he gets poor because he doesn't wag it. Anyhow, I am convinced that whenever you find a tail it is intended for protective wagging and not to sit on. I furthermore think that if a dog has a tail and won't wag it, conservative surgery should be employed to remove it,—for while the dog thereby loses his tail, there is a saving in material and in expenditure of vital energy.

Speaking of tails,—some of the arguments which are advanced in support of the aforesaid doctrines as to surgical diseases remind me of a lesson in logic. It follows: No cat has two tails. One cat has one more tail than no cat,—therefore, one cat has three tails. That would make nine cats have 27 tails. Reverse this and we get the moral. On the other hand, all sight is lost of that fixed principle that things equal to the same things are equal to each other. How in the name of common sense, to say nothing of science, can the same blood, same nervous system and the same proximate principles supply the same class of tissues while one is susceptible to primary perversion and the other is not?

We have often heard it said that possession is nine points in the law anyway, and if a disease is found in the stomach nine times out of ten, isn't it reasonable to suppose that it belongs to it? If the principle in which these teachers would instruct us are true and the same should apply to real estate, the title to no piece of property would be secure. If any part of the human body is immune from primary disease it would be interesting to know in what that immunity consists and by what process and by whose authority the order of life and the law of organic forces has been changed. And if the limitation of diseases of the Gastro-intestinal tract to secondary forms is correct or founded upon scienticfi fact, then much of the curriculum in colleges is a practice of deceit, and imposition on the students and same should be abolished.

Yet another source of medical education is the distribution of public health reports, State and Government. I need not speak of the value to the profession and benefit to the people which may accrue from these sources. But in our adoption of data furnished by these well-equipped and efficiently conducted departments of service in medicine, we may remember that in no single instance does such data have claim upon our confidence superior to that offered by a member of this Society unless that claim is based upon the discovery or establishment of a truth otherwise not in reach of the profession. In our appreciation of meritorious work and applause of distinguished achievement, and in our loyal support of every means for scientific advancement, whether official or otherwise, we cannot relax in our ideals or surrender a fact which has stood the test of long practical experience.

Federal and state initiatives are only in process of segmentation but they have in them the elements of strong directive and coercive power.

Let us hope that in the future exegencies of the social and civic relations of the profession that which is worthy and wholesome in its present status may not be sacrificed.

But it is of another feature of these reports that I wish to speak more particularly. I refer to those items which deal with medical subjects proper and the treatment of diseases. In so far as the opinions expressed in these reports accord with scientific facts and conditions as they exist in localities selected for work of investigation, so far do these opinions furnish a course of valuable instruction and the work done an example which we may follow with advantage. In so far as the opinions expressed do not accord with scientific facts and with practical conditions as they exist, so far are they capable of harm not only in point of public health, but socially, industrially, economically, and are an injustice to the people or section which is thus called into question. Instance, the theory that pellagra is due solely to poor food and unsanitary surroundings, otherwise, poverty in all its significance and results.

As militaristic as it may sound, it is nevertheless true, that we of the South need to mobilize our forces, not for purposes of aggression, not for professional aggrandizement, but that we may hold what we have and be able to contribute our quota to the sum of influence which should be exerted in behalf of the good name and well being of our state and section.

It is to be regretted that at a distance Georgia and the South are fast becoming synonymous, if they are not already so, with lynchings, hook worm, amaeba, malaria, boll weevil and illiteracy, and the last should perhaps be mentioned first because it is supposed to be associated with, if not the forerunner of all the others,—except the boll weevil,—and so far as I know this has not as yet been discovered in a human being.

The South suffers hardly more from the existence of these things than from the publicity given to their existence, and to their alleged causes. It is easy for reports of anything sensational to be exaggerated, and it is highly probably that greater significance has been attached to these infirmities of the South than their nature and extent justify. Please do not suspect that I am endeavoring to criticise the agencies which are being employed to improve the sanitation of the country and to protect the health of the people. These have been too long neglected. There are yet, as I see it, obligations to be assumed by the government, by the press, and by the medical profession, looking to the removal of those causes and conditions which underlie many of the disabilities and physical ills of the nation.

Much has been accomplished by legislation. More can be accomplished if doctors representing the true principles and purposes of the profession have sufficient voice in legislation on matters touching medical education and medical practice. Much has been accomplished by the support of the public press in forward movements of the profession. More can be accomplished if they will purge their columns of fake advertisements and spurious essays on scientific subjects. Much has been accomplished by the profession as a whole. Wonders have been worked by some individuality. More can be accomplished if there is a concerted effort towards the establishment of a standard which represents what is true in principle and right in practice. If two sides to every question will be admitted and the need of reciprocal action is recognized and carried to effect, —if the general practitioner, the specialist, and surgeon will grant each to the other the distinctness and the mutual dependence of their respective functions. We do not get anywhere by going in single file,—or by blocking the way with arbitrary rules or progression.

Where in the line is the general practitioner today? The doctor in the country,—in the city,—in the ranks,—anywhere? What has been done for him? What has been undone for him? What is before him? These are questions which I would not undertake to answer. But I venture the assertion that there are none more serious for us to consider.

